# Socio-Demographic Variation, Perceived Oral Impairment and Oral Impact on Daily Performance among Children in Saudi Arabia

**DOI:** 10.3390/ijerph16142450

**Published:** 2019-07-10

**Authors:** Saba Kassim, Hala Bakeer, Shahad Alghazy, Yara Almaghraby, Wael Sabbah, Alla Alsharif

**Affiliations:** 1 Preventive Dental Science, Taibah University Dental College & Hospital, Prince, Naif Ibn Abdulaziz, Al-Madinah Al-Munawwrah 42353, Saudi Arabia; 2 Taibah University Dental College & Hospital, Prince, Naif Ibn Abdulaziz, Al-Madinah Al-Munawwrah 42353, Saudi Arabia; 3 King’s College London, Faculty of Dentistry, Oral & Craniofacial Sciences. Denmark Hill, Bessemer Road, London SE5 9RW, UK

**Keywords:** impact, Child-Oral Impact on Daily Performance (C-OIDP), children, reliability, impairment

## Abstract

*Background:* This study aimed to describe the oral impact (estimate, severity, frequency) on daily performance (e.g., eating, speaking) and identify the potential perceived oral impairment(s) and socio-behavioral factors associated with oral impact, namely presence or absence of oral impact, among children aged 9–12 years old in Al-Madinah Al-Munawwarah, Saudi Arabia. *Methods:* A cross-sectional convenience sample of 186 children aged 9–12 years old was recruited. Sociodemographic characteristics, oral health-related behaviors, and perceived oral impairments (e.g., caries, toothache) were obtained from participants. The validated Arabic Child Oral Impact on Daily Performance (C-OIDP) inventory was used to assess oral impacts. Sample descriptive statistics and multivariable logistic regressions modeling the association between C-OIDP and explanatory variables were performed. *Results:* The mean (±SD) age of the children was 10.29 ± 1.24 years, 66.4% were from public schools, and 52% were females. At least one C-OIDP was reported by 78% of the participants. The mean C-OIDP score was 2.27 ± 1.99. Toothache was reported as a perceived impairment for almost all oral impacts and was the strongest predictor of C-OIDP. Low father income was negatively associated with C-OIDP (odds ratio (OR) = 0.24, 95% confidence interval (CI) = 0.10–0.62). Females had significantly higher odds of reporting C-OIDP than males. *Conclusions:* In this convenience sample, a high percentage of children aged 9–12 years old reported C-OIDP, which was linked to oral impairment and socio-demographic factors. Further studies, however, are required to explore the clinical, behavioral, and sociodemographic factors in relationship to C-OIDP among Saudi children in a representative sample.

## 1. Introduction

Oral health-related quality of life (OHRQoL) is a concept that captures the broader aspects of health [1] and differs from the traditional clinical indices (e.g., DMFT) measuring oral health [2]. Specifically, OHRQoL is a multidimensional construct that considers both the function and the psychological outcomes of oral diseases [3,4]. The current FDI World Dental Federation definition of oral heath emphasizes that “oral health is multifaceted and includes the ability to speak, smile, taste, touch, chew, swallow, and convey a range of emotions through facial expressions with confidence and without pain, discomfort, and disease of the craniofacial complex” [5].

A range of socio-dental indices were developed and tested to assess OHRQoL among adults and elderly population [6]. The Oral Impact on Daily Performance (OIPD) is an index that has been developed for adults [6] and was adapted to be used among children to assess the impact of oral health on quality of life in children (C-OIDP) [7]. This index was based on an explicit conceptual framework, the World Health Organization’s International Classification of Impairments, Disability, and Handicaps, which was adapted by Locker for oral health [1]. The key points of OIDP are impairments, functional limitations, pain and discomfort, disability, and handicap. Impairments are the immediate biophysical outcomes of disease, and are often assessed by clinical indicators; functions refer to the body parts’ functions (oral health function [performance] = for example, eating); pain and discomfort refer to the practical aspect of oral conditions in term of symptoms; and, finally, disability and handicap refer to the inability to perform daily activities and the related social disadvantages [8].

The C-OIDP inventory focuses on the oral impact in eight domains (eating, speaking, mouth cleaning, relaxing, emotion, smiling, studying, and social contact) with a range of impairments (e.g., toothache and tooth decay). Notably, among children, the highest impact on performance was for eating, reported by a number of studies [9,10,11,12], and the lowest reported impact was social contact and speaking [9,10,12]. Toothache was reported as the main cause of impairment and the contributor to most domains in different settings [2,10,11]. Additionally, the inventory captures both the frequency and perceived severity of the C-OIDP [11] and allows for assessing indications of individuals’ ‘care-seeking’ behavior (dentist visits) [11]; moreover, it allows for the analysis of condition-specific impacts (e.g., gingivitis) on daily performance [13,14].

The socioeconomic status (SES) disparities in C-OIDP were investigated and linked to different aspects of SES. In Sudan Nurelhuda et al. [10] reported that children from a high SES attending state schools were more likely to report C-OIDP, while in Nigeria children from a lower or middle SES (measured by a combination of mother’s education and father’s income) had increased odds of having their C-OIDP negatively affected compared with children with higher socioeconomic status [15]; finally, in France children from schools situated in deprived areas and whose mothers were immigrants and living in large families were likely to report significantly higher C-OIDP [16]. Among Brazilian children, girls were statistically significantly more likely to report C-OIDP [17], while no gender differences were found among Sudanese children [10].

Oral health-related behavior, which includes tooth-brushing frequency and dental attendance, was also investigated in relation to C-OIDP. Nurelhuda et al. [10] found no relationship between C-OIDP and tooth-brushing frequency, while Monsantofils and Bernabe [11] reported that C-OIDP was related to recent use of dental services among schoolchildren. 

The cross-cultural reliability and validation of the scale was tested and found to have adequate internal reliability (Cronbach Alpha range: 0.57–0.87) in a different setting such as Nepal, Sudan, France, and India [2,10,16,18]. The validity of the scale was assessed with the clinical findings and global self-rated oral health [16]. Notably, the validated Arabic Child version of the oral impacts on daily performance (C-OIDP) has been assessed for psychometric validity and reliability and used to assess oral health impacts in a limited Arabic setting, for example, Sudan [10]. The literature search revealed no study in Saudi Arabia (SA) that assessed oral impacts on the daily performance of children using C-OIDP, irrespective of oral-specific condition impact (e.g., malocclusion). While the application of the scale (C-OIDP) in different populations is necessary to evaluate the inventory performance [16], within the context of Saudi Arabia, it will shed light on children’s perception of oral impact on their daily performance irrespective of clinical indictors of normative needs (dentist’s assessment), and as such may highlight in epidemiological representative samples implications on many fronts including needs assessment, policy formulation, and practices’ health care provisions based on evidence [3].

Given that the C-OIDP has not been used in this region before, it was deemed important to assess the psychometric (internal consistency [Cronbach alpha]) of the C-OIDP within the context of SA.

The aim of this study was to describe the oral impact (estimate, severity, frequency) on daily performance (e.g., eating, speaking) and identify the potential perceived oral impairment(s) and socio-behavioral factors associated with oral impact, namely presence or absence of oral impact, among children aged 9–12 years old in Al-Madinah Al-Munawwarah, Saudi Arabia. 

Our research questions were as follows: (1) Do the data from this survey confirm the psychometric characteristics (internal consistency) of the C-OIDP? (2) What are the aspects of C-OIDP (e.g., Severity)? (3) Does the C-OIDP vary according to socioeconomic status, self-reported oral-health-related behavior (e.g., dentist visits), and the perceived oral impairments of the child?

## 2. Materials and Methods

### 2.1. Study Design, Setting, and Participants’ Recruitment

A cross-sectional, community-based study recruited a convenience sample of 186 children in one of the biggest shopping malls during the school midterm holiday in February 2017. This mall, which hosted the annual Taibah University Dental College and Hospital Outreach Program, was in the city of Al-Madinah Al-Munawwarah, SA. This annual program is part of the graduate dental students’ curriculum and runs for one month, providing services to the local community that include dental education (e.g., oral hygiene instructions), oral examination, referral, and distribution of free toothpaste and toothbrushes. We used this strategy (time and setting) to address the aims of the study, that is, this mall is one of the biggest in the city and is frequented during the whole year, but especially during school holidays, by local residents.

A consecutive sample of children aged 9–12 years who self-reported residing and attending school in 2016–2017 in Madinah, SA, accompanied by an adult (e.g., parents, cousins), were invited into the study during their visit to the Taibah University Dental College and Hospital Outreach program in the mall. Children who had systemic diseases and were taking medication (e.g., antibiotics) were excluded, as this might contribute to their poor oral health [8].

### 2.2. Data Collection and Variables

Structured face-to-face interviews were conducted to collect information on child socio-demographic and socioeconomic status, namely, age, gender, nationality (Saudi and non-Saudi), type of school (public or private), and locality of residency to determine the level of the area’s deprivation (poor, medium, or high). In addition, children were asked to report the level of education and occupation of their parents, which was used as a proxy of monthly income (low or high).

As for the C-OIDP, the validated Arabic version of the Child Oral Impact on Daily Performance (C-OIDP) questionnaire was administered [19]. The child was asked to respond whether oral health affected him/her in one of the eight performance domains (physical [eating, speaking, mouth cleaning, and physical activities], psychological [relaxing/sleeping, emotion, and smiling], social [studying and social contact]) that could be related to their dental or mouth condition [10,19,20].

If the child reported oral impact on any of the aforementioned daily performance, follow-up questions about the frequency and severity of the impact were asked. Both the frequency and severity of impacts were scored on a three-point Likert scale (1–3). The frequency scores were as follows: (1) once or twice a month, (2) once or twice a week, and (3) three times or more a week. As for the severity score, these were (1) mild, (2) moderate, and (3) severe. Finally, the children were asked to choose from a list of 16 impairments one or more oral conditions they perceived as the cause of the impact(s). These conditions were toothache, sensitive teeth, tooth decay (hole in teeth), exfoliating primary teeth, tooth space (due to a non-erupted permanent tooth), fractured permanent tooth, color of tooth, shape or size of tooth, position of tooth, bleeding gum, swollen gum, calculus, oral ulcers, bad breath, deformity of mouth or face, erupting permanent tooth, and missing permanent tooth [19].

The C-OIDP extent was calculated according to the performance in the eight domains affected by impacts, so the range was from 0 to 8. If the child reported no impact, then a 0 score was assigned, versus 1 for at least one impact. As for oral health-related behaviors, children’s tooth-brushing frequency and use of dental services were assessed. The latter was self-reported and measured on a five-point response scale (in the last three months, more than three months ago but less than 12 months, more than 12 months but <24 months, more than 24 months, or never) [11], whereas the former was on a six-point scale (more than once a day, once a day, weekly, irregularly, not at all, or I do not brush my teeth). Four dentists (two males and two females) were assigned and trained to carry out the interviews. 

### 2.3. Ethical Clearance

The study protocol was granted ethical approval (TUCDREC/20I70305/Bakeer) after review by the Research Ethics Committee of Taibah University, Al-Madinah. The study was conducted in accordance with the principles of the World Medical Association of Helsinki. The consent of the child’s guardian was obtained alongside child assent (willingness to participate) prior to the interviews. Participation in the study was voluntary and every questionnaire was anonymous and coded to optimize the confidentiality of the obtained information. Interviews were conducted in a confidential atmosphere.

### 2.4. Statistical Analysis

Descriptive statistics were executed to report sample characteristics and the frequency and severity of C-OIDP impacts. This analysis included mean and standard deviation (SD) for continuous variables (e.g., age) and frequency with percentages (F [%]) for qualitative variables (e.g., gender). A number of participants were not aware of their mother’s and father’s level of education attained or the parents were not willing to disclose it. Only the father’s occupation, which was used as a proxy for income, was used in the analysis. The psychometric properties of the C-OIDP internal consistency reliability test (Cronbach’s alpha [α] and α if an item was deleted) were assessed. The outcome of interest (dependent variable, DV) was oral impact on daily performance and a dichotomous indicator of C-OIDP was used based on reporting at least one impact (no impact = 0 and impact = 1 or more). A chi-squared test was performed to assess the factors associated with the dependent variable. Multivariable logistic regressions were run to determine the potential predictors of C-OIDP. The *p*-value was set at *p* < 0.05 for both bivariate and multivariable analyses. Given that we used a convenience sample recruited from the mall, no a priori sample size was calculated. However, in the absence of a similar study in SA and based on the prevalence of oral impact in Sudan [10], the post hoc power calculation for this study was 100%.

## 3. Results

### 3.1. Background Characteristics, Oral Health-Related Behaviors, and Perceived Oral Impairments

The mean (±SD) age of participating children (186) was 10.29 ± 1.24 years, and slightly over half of the participants (97, [52.2%]) were female. Table 1 shows the other characteristics of the sample. As for oral-related behavior, 78 (41.9%) attended the dentist within the last year and 62 (33.3%) had attended the dentist more than two years ago, or had never attended. Brushing teeth more than once a day was reported by 75 (40.3%), while 28 (15.1%) reported that they did not brush their teeth. As for accessibility to health care, of the sample, only 103 (55.4%) reported the proximity of heath care to their homes.

Perceived oral impairments that contributed to C-OIDP were significantly higher among female participants than males. Namely, sensitive teeth (47% female vs. 13% male, *p* < 0.001), tooth decay (62% female vs. 39% male, *p* = 0.002), exfoliating primary teeth (51% female vs. 33% male, *p* = 0.013), tooth space due to non-erupted teeth (36% female vs. 20% male, *p* = 0.017), position of teeth (53% female vs. 22% male, *p* < 0.001), bleeding gums (56% female vs. 33% male, *p* = 0.002), calculus (18% female vs. 7% male, *p* = 0.026), oral ulcers (29% female vs. 8% male, *p* < 0.001), and eruption of permanent teeth (45% female vs. 33% male, *p* = 0.075).

### 3.2. Psychometric Properties and Responses for C-OIDP (Estimate, Frequency, Severity, and Extent)

To address our first and second research questions, the psychometric characteristics of C-OIDP were evaluated. The internal reliability Cronbach’s alpha (α) for C-OIDP was 0.70 and the range of α if an item was deleted was between 0.640 and 0.720. A drop of α to 0.640 was observed when the domain of ‘Eating’ was deleted and it increased to 0.720 when the domain of ‘Smiling’ deleted. The overall estimate of children with oral impacts (at least one impact) was 78%. Table 2 shows that the impact of oral condition on the daily performance of children’s eating during the last three months was the most reported (45.7%). Children perceived that toothache was the impairment contributing most to seven impacts (eating [67%], speaking [69%], mouth cleaning [53%], relaxing [70%], emotion [73%], study [90%], and contact with people [68%]). Poorly positioned and exfoliated teeth were equally (47%) perceived as the impairments that most impacted smiling. The frequency and severity were high among children reporting oral impact on eating (Table 2). Of the 85 (45.7%) participants who reported an impact on eating, the frequency of oral impact was more or less weekly, as reported by 48 (56.5%), while severity was reported by 36 (42%).

**Figure 1:**
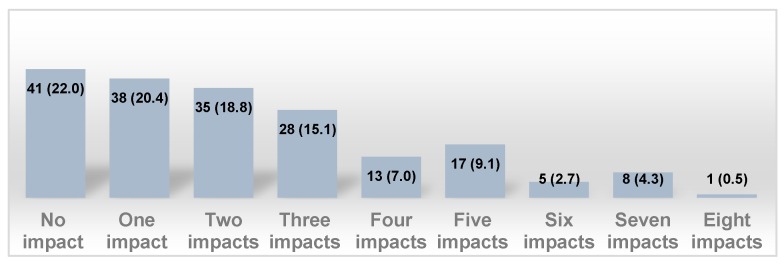
Frequency distribution and percentages (F [%]) of number of impact performance experienced among children in Madinah, Saudi Arabia (SA) (*n* = 186).

The overall mean value of the extent of the impact performance experienced among this study sample was 2.27 ± 1.99. Figure 1 shows that 73 (39.2%) reported one to two impacts.

### 3.3. Factors Associated with C-OIDP

As for the third question of our research, the bivariate analysis showed that age, area level (poor, medium, rich), nationality (Saudi vs. non-Saudi), and school type (public or private) were not associated with C-OIDP at *p* < 0.05; likewise with tooth-brushing, proximity of health care from the child’s home, and dental attendance. However, female participants reported significantly more oral impact on daily performance compared with males (83 [57.6%] vs. 61 [42.4%], *p* = 0.006). Participating children whose parents had a low income were significantly less likely to report C-OIDP compared with their counterpart children whose parents had a high income (31 [67.4%] vs. 111 [82.2%], *p* = 0.035). Additionally, participants who reported toothache were significantly more likely to report C-OIDP then their counterpart children who reported not having toothache (101 [91.9] vs. 43 [65.6%], *p* < 0.001). Multivariable modeling (Table 3) revealed that females and children with toothache were 3.27 times (95% confidence interval (CI) = 1.38–7.69, *p* = 0.007) and 10.65 times (95% CI = 4.23–26.84, *p* = 001) more likely to report oral impacts on daily performance, respectively. However, children whose parents had a low income were protected from oral impacts on daily performance (odds ratio (OR) = 0.24, 95% CI = 0.10–0.62, *p* = 0.003).

## 4. Discussion

The aim of this study was to describe the oral impact (estimate, severity, frequency) on daily performance (e.g., eating, speaking) and identify the potential perceived oral impairment(s) and socio-behavioral factors associated with oral impact, namely presence or absence of oral impact, among children aged 9–12 years old in Al-Madinah Al-Munawwarah, Saudi Arabia. In addition, we further assessed the psychometric (internal consistency ([Cronbach alpha])) of the C-OIDP within the context of SA.

The C-OIDP inventory in this culture (SA) showed acceptable reliability (Cronbach alpha, 0.70), as reported elsewhere [7,10,21]. The estimate of oral impact on daily performance among this study sample was within the range of other studies [11,16]. In addition, the extent and average number of impacts aligned with other studies conducted in Peru and India [11,18]. The main impact of C-OIDP was found for the eating domain and the main perceived contributor to this impact was toothache; this concurs with other studies [10,11,18], as well as the severity of the impact on eating [9,18].

As for the socio-demographic and behavioral factors in relationship to C-OIDP, this study suggested that C-OIDP was less among deprived children, though this is a paradox as a number of studies showed that deprived children had more OIDPs [15,16]. Our findings aligned with a study in Sudan [10], which found that state school children from a high SES were more likely to report C-OIDP and attributed that to their SES, which made them more knowledgeable and aware of oral impacts compared with other children. Within the context of this study, family financial constraints could have played a protective factor, that is, children could have had less access to oral health risks such as sweets and fizzy drinks and as such had fewer OIDPs. Alternatively, the deprived social environment of the study’s children, manifested in the low family income, might not prioritize oral health and as such acted as a barrier to children being knowledgeable about oral health. Notably, the results should be interpreted cautiously because of the sampling inadequacy (convenience sampling).

However, the modulation of gender with C-OIDP showed that females were approximately three times more likely to report C-OIDP compared with males; this aligned with the findings reported by Peres et al. [17], but was in contrast to those reported by Nurelhuda et al. [10], who found no difference between male and female students in C-OIDP. This could be attributed to the cultural context of the study. Access to oral health care among females in SA is often delayed because of culture. This was evident in this study, with female participants perceiving more oral health impairments (Section 3.1) compared with males. Oral health-related behaviors such as frequency of tooth-brushing and dental visits were not found to be related to C-OIDP, the former findings aligned with a study conducted in the same region (Sudan) [10]. However, Monsantofils and Bernabe [11] reported an association of dental attendance with C-OIDP.

Several limitations should be acknowledged in this study. The sample was not representative of all children and was based on convenience, so the study findings are not widely generalizable. Causality cannot be inferred as the nature of the study was cross-sectional. We have used the father’s occupation as a proxy for family income; this may prone to bias, however, as the setting and timing of the study prevented more information from being obtained. Information about private school type was not obtained, that is, schools may have different levels. Finally, the stability of the scale (test–retest) of the C-OIDP was not assessed because of a number of constraints, that is, the nature of the study setting (a mall) and convenience sampling of the children used. This study highlights the impacts of perceived oral impairments among primary school children in Al-Madinah, Saudi Arabia. Although the findings of the study did not show a relationship between tooth-brushing and dental attendance, investment in oral health promotion activities and improvement in access to dental services are likely to improve the impact on children’s daily performance in this region. Notably, this first requires the application of the C-OIDP in a representative epidemiological study that considers the modulation of the clinical, behavioral, and sociodemographic factors of the children residing in SA.

## 5. Conclusions

The C-OIDP was found to be reliable and a significant number of children reported oral health impacts on at least one aspect of their daily performance. Eating was most impacted, with toothache as a common perceived impairment. Oral health impacts on daily performance were significantly associated with perceived oral impairment and sociodemographic factors. However, further studies are required to explore the clinical, behavioral, and sociodemographic factors in relationship to C-OIDP in a representative sample of Saudi children.

## Figures and Tables

**Table 1:** Socio-demographic and oral health-related behaviors among children in Madinah, Saudi Arabia (SA) (*n* = 186).

Variables	Mean ± SD or F (%)
**Age (mean)**	10.29 ± 1.24
Nationality *	
Saudi	120 (64.5)
Non-Saudi	65 (34.9)
School type *	
Private	37 (19.9)
Government (State)	137 (73.7)
Residency area level *	
Poor	33 (17.7)
Medium	109 (58.6)
Rich	27 (14.5)
Father’s income *	
High	135 (72.6)
Low	46 (24.7)
**Dental attendance**	
In the last three months	64 (34.4)
More than three months ago but less than 12 months	14 (7.5)
More than 12 months ago but <24 months	46 (24.7)
More than 24 months ago or never	62 (33.3)
Tooth-brushing *	
More than once a day	75 (40.3)
Once a day	37 (19.9)
More than once a week	10 (5.4)
Once a week	4 (2.2)
Irregularly	31 (16.7)
Not at all; I do not brush my teeth	28 (15.1)

* Does not include non-responses.

**Table 2:** The Oral Impact on Daily Performance (estimate, frequency, and severity) among children in Madinah, SA (*n* = 186).

Variables	Eating	Speaking	Mouth Cleaning	Relaxing	Emotion	Smiling	Study	Contact
Estimate of C-OIDP on each of the eight domains								
C-OIDP F (%) *								
Yes	85 (45.7)	29 (15.6)	64 (34.4)	69 (37.1)	62 (33.3)	63 (33.9)	29 (15.6)	22 (11.3)
No	101 (54.3)	157 (84.4)	122 (65.6)	117 (62.9)	124 (66.7)	123 (66.1)	157 (84.4)	164 (88.2)
Frequency (monthly or weekly) of the impact among those who reported an impact								
≤2 per month	37 (43.5)	16 (55.2)	25 (39.1)	33 (47.8)	34 (54.8)	23 (36.5)	18 (62.1)	14 (63.6)
≤2 per week	26 (30.6)	9 (31.0)	18 (28.1)	20 (29.0)	17 (27.4)	20 (31.7)	9 (31.0)	4 (18.2)
≥3 per week	22 (25.9)	4 (13.8)	21(32.8)	16 (23.2)	11 (17.7)	20 (31.7)	2 (6.9)	4 (18.2)
Severity of the impact among those who reported an impact								
Mild	16 (18.8)	8 (27.6)	21 (32.8)	20 (29.0)	22 (35.5)	28 (44.4)	10 (34.5)	8 (36.4)
Moderate	33 (38.8)	10 (34.5)	25 (39.1)	24 (34.8)	21 (33.9)	19 (30.2)	10 (34.5)	4 (18.2)
Severe	36 (42.4)	11 (37.9)	18 (28.1)	25 (36.2)	19 (30.6)	16 (25.4)	9 (31.0)	10 (45.5)

* F (%) = frequency with percentages.

**Table 3:** Results of multivariable logistic regression of the association between C-OIDP **^∞^** and explanatory variables among children in Madinah, SA (*n* = 186).

Explanatory Variables	B	Wald	OR (95% CI) *	*p*-Value
**Gender**				
Male			1	
Female	1.184	7.337	3.27 (1.39–7.69)	0.007
**Father’s income**				
High			1	
Low	−1.415	8.815	0.24 (0.10–0.62)	0.003
**Toothache**				
No			1	
Yes	2.366	25.197	10.65 (4.23–26.84)	0.001

∞C-OIDP = Child Oral Impact on Daily Performance; no impact = 0, impact = ≥1; * OR (95% CI) = odd ratios and 95% confidence interval.
